# Functional MRI using robotic MRI compatible devices for monitoring rehabilitation from chronic stroke in the molecular medicine era (Review)

**DOI:** 10.3892/ijmm.2012.942

**Published:** 2012-03-15

**Authors:** LOUKAS G. ASTRAKAS, SYED HASSAN ABBAS NAQVI, BABAK KATEB, A. ARIA TZIKA

**Affiliations:** 1 Department of Surgery, NMR Surgical Laboratory, Massachusetts General Hospital and Shriners Burn Institute, Harvard Medical School, Boston, MA 02114, USA; 2 Department of Medical Physics, University of Ioannina, Ioannina 45110, Greece; 3 Department of Radiology, Athinoula A. Martinos Center of Biomedical Imaging, Massachusetts General Hospital, Boston, MA 02114, USA; 4 Brain Mapping Foundation, West Hollywood, CA 90046, USA; 5 Dow Medical College, Dow University of Health Sciences, Karachi 74200, Pakistan; 6 Society for Brain Mapping and Therapeutics (SBMT), West Hollywood, CA 90048, USA; 7 National Center for Nano-Bio-Electronics (NCNBE), Moffett Field, CA 94035-0023, USA; 8 Department of Neurosurgery, Maxine Dunitz Neurosurgical Institute, Cedars Sinai Medical Center, Los Angeles, CA 90048, USA

**Keywords:** stroke, stroke biomarkers, rehabilitation, functional MR imaging, robotic MRI compatible devices

## Abstract

The number of individuals suffering from stroke is increasing daily, and its consequences are a major contributor to invalidity in today’s society. Stroke rehabilitation is relatively new, having been hampered from the longstanding view that lost functions were not recoverable. Nowadays, robotic devices, which aid by stimulating brain plasticity, can assist in restoring movement compromised by stroke-induced pathological changes in the brain which can be monitored by MRI. Multiparametric magnetic resonance imaging (MRI) of stroke patients participating in a training program with a novel Magnetic Resonance Compatible Hand-Induced Robotic Device (MR_CHIROD) could yield a promising biomarker that, ultimately, will enhance our ability to advance hand motor recovery following chronic stroke. Using state-of-the art MRI in conjunction with MR_CHIROD-assisted therapy can provide novel biomarkers for stroke patient rehabilitation extracted by a meta-analysis of data. Successful completion of such studies may provide a ground breaking method for the future evaluation of stroke rehabilitation therapies. Their results will attest to the effectiveness of using MR-compatible hand devices with MRI to provide accurate monitoring during rehabilitative therapy. Furthermore, such results may identify biomarkers of brain plasticity that can be monitored during stroke patient rehabilitation. The potential benefit for chronic stroke patients is that rehabilitation may become possible for a longer period of time after stroke than previously thought, unveiling motor skill improvements possible even after six months due to retained brain plasticity.

## 1. Introduction

Stroke is a major cause of morbidity and invalidity in modern society. The most common form occurs when an obstructed blood vessel prevents blood flow to part of the brain, depriving cells of oxygen resulting in loss of motor control. Stroke afflicts approx. 795,000 people each year and is the most prevailing cause of severe, long term disability and the cost of their care is among the fastest-growing expenses for medicare (1).

Around 80–90% of stroke survivors exhibit motor weakness and 40–50% experience sensory dysfunction (2). The likelihood of improvement after stroke varies with the nature and severity of the initial deficit. For example, 6 months after stroke about 65% of patients cannot incorporate the affected hand into their usual activities. Poor upper-extremity outcomes, defined by no hand movement or only slight finger flexion with no opening 4 weeks post-stroke, are common after a hemispheric infarction with considerable damage to the corticospinal tract (3). Stroke survivors generally achieve some degree of physical recovery within 3 months of the insult. However, only 25% achieve recovery of function to the level of everyday physical functioning seen in community-matched persons who have not had a stroke (4). In particular, cognitively impaired stroke patients experience poor recovery of activities necessary for independent daily living (5). Understandably, quality of life tends to be higher among patients with better functioning than among those with worse functioning (6).

Functional recovery after stroke can be attributed to brain plasticity, which has the amazing ability to adjust itself by forming new connections between brain neurons (7,8). Both animal and human studies on stroke recovery have correlated functional reinstatement with brain reorganization (8–15). For example, intracortical micro-stimulation mapping in monkeys has demonstrated that shifts of hand representations occur following focal ischemic lesions in the sensorimotor cortex (16,17). In addition, brain imaging studies in chronic stroke patients have shown that plastic changes can occur, including enhanced bilateral activation of the sensorimotor cortex, increased activity in secondary or higher order sensorimotor areas and recruitment of additional cortical areas during performance of a hand sensorimotor task (12). A clear causal link between cerebral reorganization and functional recovery has been suggested (13,15) and post-stroke care with the aim of reducing long-term disability continues to advance (18). Unfortunately, objective evaluation of the specific effects of rehabilitation is technically challenging (19). Substantially more information regarding the events of post-stroke functional recovery is needed to provide a firm neurobiological foundation for evaluation strategies.

Recent studies that have examined the time course of motor recovery after stroke have found that the greatest gains in motor function occur within the first month of recovery, with some additional improvement being observed up to 6 months post-stroke (20,21). Improvement, especially during the first few weeks after a stroke, is attributed to the recovery of neurotransmission in spared tissue near and remote from an infarct or hemorrhage (22,23). Stroke patients then generally exhibit a plateau of functional recovery and little or no further progress is expected beyond the 6- to 12-month post-stroke period. This recovery profile has traditionally been attributed to the presumption that the central nervous system has a limited capacity for reorganization and improvement in function. However, recent studies of chronic post-stroke motor impairments have shown that intensive therapeutic interventions lead to significant improvements in cortical reorganization and motor function in persons tested more than one year post-stroke (24–31). Empirical evidence suggests that a plateau in motor recovery after stroke may be related to the timing and intensity of stroke rehabilitation services. Ideally, rehabilitation should occur as soon as the diagnosis of stroke is established and the individual is clear of any life-threatening problems (32). At any time after a stroke, however, cognitive, language and motor skills may improve as a result of the cerebral processes involved in ordinary learning. This experience-induced neuroplasticity includes greater excitability and recruitment of the neurons in both hemispheres of the brain, sprouting of dendrites that communicate with other neurons and strengthening of synaptic connections. We predicate that an improved understanding of the mechanisms underlying functional recovery in chronic stroke patients will help researchers design effective neurorehabilitative strategies to further improve patients’ recovery.

## 2. Functional neuroimaging methods for evaluating stroke

Brain-mapping techniques have proven to be essential in comprehending the molecular, cellular and functional mechanisms of recovery after stroke (33). The currently available non-invasive functional methods for evaluating stroke are based on hemodynamic or electrophysiological principles. Techniques based on hemodynamic principles include functional magnetic resonance imaging (fMRI), positron emission tomography (PET), single-photon emission computed tomography (SPECT) and near-infrared spectroscopy (NIRS). Electrophysiological techniques include electroencephalography (EEG), magnetoencephalography (MEG) and transcranial magnetic stimulation (TMS). Reviews of each particular technique are available in the literature (34–41) and a recent integrative review of all these techniques from the viewpoint of clinical neurophysiology was recently published (42). Importantly, only fMRI and PET allow imaging of deep brain structures such as the basal ganglia. The other technologies applied in the characterization of force control after stroke only permit characterization of the cerebral cortex and TMS can only be used to map regions capable of evoking motor responses.

Given the varying features of the functional neuroimaging methods available, a combination of two or more complementary techniques may provide greater information than any single technique. The combination of one method based on hemodynamic principles and one based on electrophysiological principles would be preferable. To this end, several investigators have used fMRI in combination with EEG (39,40,43–45). By itself, EEG is responsible for recording different brain wave frequencies and patterns (46). However, when EEG is recorded simultaneously with fMRI, artifacts arising from the MRI scanner gradients interfere with the EEG and despite technical advances (47), artifact elimination still remains problematic. Nevertheless, the simultaneous recording of EEG in the MR scanning room is especially favorable for the presurgical evaluation of patients with medically intractable partial epilepsy (40,48).

## 3. Functional MR imaging

The underlying principle of fMRI is to develop images of the brain, which reflect brain tissue hemodynamics and demonstrates the concept that neuronal activation is accompanied by increased regional blood flow. A valuable advantage of fMRI is that brain anatomy and blood flow can be measured simultaneously. Spatial resolution is typically in the range of 1–5 mm and temporal resolution is measured in seconds. The fMRI techniques used for mapping brain activity are blood oxygenation level-dependent (BOLD) imaging and perfusion imaging. BOLD imaging, which is the most widely applied, uses endogenous deoxyhemoglobin as a contrast source. Through this method, neuronal activation is inferred from small, local MR signal alterations proportional to hemodynamically induced changes in net deoxyhemoglobin concentration caused by task-related increases in neuronal metabolism (49). We can observe this when cortical activation causes alterations of blood oxygenation, which create changes in microscopic susceptibility measured using T2^*^-weighted sequences. The application of fast MR scan techniques, such as FLASH (50–52), EPI (53–55), spiral scan (56–59) and PRESTO (60–62), for fMRI has improved temporal resolution and allowed the detection of transient changes in deoxyhemoglobin concentration following presentation of brief stimuli (63–67). An alternative, relatively new fMRI approach, i.e., resting state fMRI (rs-fMRI) may allow greater assessment of changes in organization of whole functional networks (68). On the other hand, arterial spin labeling (ASL) MRI, which permits the non-invasive quantification of regional brain tissue perfused with labeled, inflowing arterial protons (69), has been used to detect evoked changes in neuronal activity (70–73). Many researchers have compared BOLD and perfusion fMRI (74–77) and reported that BOLD imaging is generally faster, more sensitive and has better temporal resolution than perfusion imaging. On the other hand, the ASL techniques are associated with fewer vascular artifacts, more closely reflect neuronal activity, and generate data that do not require temporal autocorrelations. Recently, new sequences capable of measuring BOLD contrast and perfusion simultaneously have been developed (78,79).

## 4. Functional neuroimaging of cerebral reorganization in stroke recovery

Brain imaging techniques can illustrate the plastic potential of the adult human brain in healthy subjects and in stroke patients (12). Brain function changes may occur in both the stroke and non-stroke hemisphere. Functional neuroimaging studies have shown the evolution of cerebral activity in both hemispheres as patients’ skills improve with training and experience (80). Serial studies using non-invasive imaging techniques have the potential to contribute to preclinical therapy evaluation by providing insights into the timing and site of stroke-relevant changes. Several techniques, with various advantages and limitations, have been used to map changes during or after stroke recovery, including positron emission tomography (PET), fMRI and transcranial magnetic stimulation (TMS) (81). Motor and language recovery are typically assessed, likely due to the ease with which they can be measured. Seminal studies from the laboratory of Richard Frackowiak (82–84) and subsequent results reported by others (85–93) have provided insights into brain reorganization following brain injury. In addition to the above techniques, single-photon emission computed tomography, near infrared spectroscopy, high-resolution electroencephalography (EEG) and magnetoencephalography have recently begun to be applied in the clinic for stroke patient evaluation. Moreover, there is reported evidence of structural plasticity co-localized with areas exhibiting functional plasticity in the human brain after stroke (94), and there has been recently provided direct evidence of brain plasticity in chronic stroke patients (95,96). Since there is limited knowledge explaining why some patients recover relatively completely, while others do not, it has been suggested that insights from functional neuroimaging studies may improve our ability to predict recovery and guide selection of individuals likely to benefit from particular treatment courses (97). Eliassen and colleagues have provided a recent review of functional neuroimaging post-stroke recovery (33).

The increased spatial resolution of fMRI has clarified neuroanatomical relationships that appeared equivocal with other methods (98). Cao *et al* (99) first applied fMRI to stroke recovery in a study of teenage patients who had suffered perinatal infarcts. Several stroke recovery studies have demonstrated that fMRI can, with high sensitivity, assess cortical and subcortical reorganization after stroke (84,98,100–107). Widespread early activation responses in the unaffected hemisphere after stroke do not appear to be directly associated with functional recovery (13,15,107) and restored function may more directly correlate with gradual reinstatement of representational neuronal fields and/or recruitment of perilesional networks (13,15). Johansen-Berg *et al* demonstrated activation of cortical motor areas in the unaffected hemisphere during movement of paretic hands and suggested that this activation reflects an adaptive response to stroke injury in associated brain (107). These findings of biphasic cerebral rearrangements underlying functional reinstatement provide potential insights into the neurophysiological mechanisms activated by neurorehabilitative therapies, and could contribute to optimizing existing therapeutic approaches.

General conclusions regarding the influence of interventions on activation patterns are similar for both upper and lower extremity (gait) movement (though these two categories of movement are quite distinct). The specific regions identified with each type of movement differ because of the differing motor task properties and imaging modalities used in the assessment of each. Five studies examining repetitive task rehabilitation interventions have demonstrated changes in brain activation post-intervention that were associated with improved upper limb (108) or lower limb (109–111) motor performance after stroke. Some discrepancies in the brain activation patterns observed using the same intervention were apparent. Specifically, Dong *et al* (108) showed reduction in activation of the undamaged contralesional hemisphere after a constraint-induced movement therapy (CIMT) intervention, whereas Kopp *et al* (112) found that the contralesional hemisphere was recruited more after a CIMT intervention. Because the sample sizes were small in both studies, it is difficult to determine whether these differences are due to stroke severity, stroke chronicity, lesion location or some other combination of factors. Moreover, different imaging modalities were used in these two studies (EEG vs. fMRI), making a direct comparison of results strained, if not impossible. Generally, interpretation of fMRI in stroke patients is an arduous task. Particular consideration should be given to baseline circulatory status. For example, major decreases in BOLD signal have been demonstrated in stroke patients as a result of decreases in cerebral blood oxygenation during neuronal activation, commensurate to the degree of ischemia (113). In addition, extra- or intracranial artery diseases influence negatively the neurovascular coupling and the cerebrovascular reserve capacity and consequently decrease the BOLD signal (114).

The ability to determine how patterns of brain activation shift with improved motor performance has great implications for ongoing research informing the development of therapies intended to manipulate brain reorganization. For example, repetitive TMS applied to the cortex is being examined as a tool to promote cortical plasticity in individuals who have suffered strokes (115) and could be used with other rehabilitation therapies to further promote functional motor programs (116). In addition, as has been shown in CIMT studies (108,112), that consideration of whether and how interventions shift activation in brain regions associated with the control of force will be critical for determining the efficacy of new treatment approaches. However, it appears that some degree of sparing of primary motor areas and associated network of secondary regions is a prerequisite for these types of interventions to produce functional movement and result in treatment success. Thus, the use of fMRI and other non-invasive neuroimaging techniques to identify residual anatomical areas and their relative contribution to functional movement may aid in determining which persons with stroke will benefit greatest from such treatments.

## 5. Robotic devices for patient rehabilitation

Rehabilitation is an active and dynamic process that assists a disabled person to gain the required knowledge and skills to maximize their own physical, mental and social function (117). During recent years rehabilitation robotics has emerged as a highly active collaborative research area between the robotics and medical rehabilitation communities (28,118–122). Robots provide both movement controllability and measurement reliability; making them ideal instruments to help neurologists and therapists address the challenges facing neurorehabilitation (123). Recent technological advances have made it possible to safely employ robotic devices in the intensive rehabilitative therapy of individuals with mild to severe motor impairments following neurological injury (124). Improved technology has allowed advancements that will enable these future versions of these devices to apply tension resisting the motion of joints. In addition to providing new options for treatment, this technology may enhance our understanding of the mechanisms that underlie the recovery of motor function and neural reorganization after stroke. Robot-assisted therapy has been shown to benefit patients during neurological recovery (30,125–134). Specifically, persons who received robotic therapy exhibited improved gain in motor coordination and muscle strength of the exercised shoulder and elbow relative to control subjects (135). Furthermore, Volpe *et al* (127) reported that these improvements were sustained over a three-year period following inpatient discharge from the hospital. Although some concerns remain, most physicians show interest in purchasing robotic devices; however the biggest challenge for robotics in rehabilitation is the limited amount of scientific evidence elucidating appropriate therapy for motor dysfunction after a stroke (136). One suggestion is that therapy is more effective when it involves several functional tasks assisted by a virtual reality environment (137). Indeed robotics can be combined with virtual reality for motor rehabilitation (137).

Most MR-compatible devices are sensing systems, such as those that quantify force exerted by subjects using their hands or wrists (138–140). For example, the device by Riener *et al* (141) uses optical force sensors to measure handgrip strength. These systems are not capable of applying any force and are used only for monitoring. The number of actuated robotic systems for MRI is small (142,143) and most of these systems have a very low range of force or motion.

Post-stroke therapy can significantly improve recovery and reduce long-term disability (18), but objective methods for evaluating the specific effects of rehabilitation are needed. While the findings of many studies have supported the hypothesis that functional brain changes accompany therapy-mediated improvements in motor skills (14,27,93,106,112,144), the spatial specificity of current evaluation methods is inadequate to allow clear neuroanatomical localization of functional changes. Initial clinical studies of robotic therapy suggest that robotic devices not only improve motor recovery, but are also at least as effective as training with physical therapists alone to restore arm function and gait. While investigators have not yet conclusively demonstrated that motorized therapy is superior to comparable non-motorized therapy, the incremental improvements in clinical scales following intensive robotic therapy, although small, are statistically significant and certainly meaningful to patients.

## 6. The Magnetic Resonance Compatible Hand-Induced Robotic Device approach

Recently an electrorheological fluid (ERF)-based actuated robotic system for MRI (MR_CHIROD) has been built and tested it in adult volunteers and stroke patients for accurate, sensitive and specific information about the effectiveness of rehabilitation therapy beyond the traditional paradigms (145–148). MR_CHIROD is unique in its ability to apply resistive force to the hand over a large dynamic range (up to 200 N) and monitor applied force, as well as in its physical characteristics of being compact and light.

MR_CHIROD fills the existing need for available rehabilitation robotic device that can be used with an MR scanner to measure both force and torque while applying computer-controlled time-varying force and torque. MR_CHIROD is novel in that it employs an unconventional type of semi-active actuation, namely electrorheostatical fluids (ERFs) consisting of dielectric microspheres dispersed in an insulating liquid, that make it MR-compatible (149). Although MR_CHIROD is small, light and inexpensive, it is capable of producing large, computer-controlled, time-varying resistive torque. Unlike previously described devices (124,150), MR_CHIROD is the first ERF-based device that has been demonstrated to function in conjunction with fMRI for online brain mapping in chronic stroke patients (95,149). Importantly, MR_CHIROD is capable of limiting and controlling a number of factors that affect its operation, making it particularly useful for home-based training given the low level of expert clinical support in the home environment which can be accompanied by low extrinsic motivation. Relative to physical therapist-facilitated training in a hospitals, home training is less expensive and more convenient, making it easier for patients to adhere to daily therapy. MR_CHIROD can be re-engineered to improve the cost-to-benefit ratio and therapy effectiveness by providing autonomous and recordable training programs with extrinsic motivation through virtual reality technology. Virtual reality can engage patients, increase their attention during the task and improve motivation, thus increasing the effectiveness of rehabilitation (151).

The rationale for building and using an MR-compatible hand robot is that while robotic therapy has been shown to improve arm motor function after stroke with few exceptions (119,152), these efforts have not been focused on the hand (135). Given the central role that hand movements normally play in people’s daily lives, more attention should be devoted to the study of rehabilitation of hand motor function after stroke. Since a major issue in hand motor therapy is how to best restore function, interventions emphasizing intense, active, repetitive movement are of high value. These interventions increase strength, accuracy and functional use when applied to subjects with paresis due to stroke. For chronic stroke patients who are in advanced stages of therapy, rehabilitation should be aimed at returning an individual to normal activities and thus incorporate resistance exercises intended to support renewed development of muscle strength. MR_CHIROD approach provides such therapy and it was motivated first by the paucity of efforts that have been made thus far concerning robotic developments for the hand and second by the unique combination of features that make our MR-compatible hand robot hold promise for enhancing traditional post-stroke therapy. These features include: i) it can provide therapy via exercises and control of the hand for long time periods in a consistent and precise manner; ii) it can be programmed for real-time adjustments of the applied force and motion according to the desired force of contraction; iii) it can measure and record performance parameters through a computer; and iv) it can be adjusted to perform with only remote human control. The latter feature extends the promise of controlling the hand robots in the MRI suite from a distance without the operator having to interrupt MRI scanning, which would improve data quality. Finally, the most notable difference between MR_CHIROD and the new breed of rehabilitation devices in use is its highly adaptive, versatile and reprogrammable nature. Computer control is intrinsic to our design and is a central theme behind this robot’s operation, making it a highly effective tool. Thus, the advantages of our advanced MR-compatible hand robot are low-cost, portability, real-time abilities and versatility.

After the first generation of MR_CHIROD (145–148) two new designs have been developed; one with rotary damper/brakes and another with linear damper/brakes. The linear damper/brake version was chosen for fabrication because of its simplicity and lower cost (153). The ERF properties and performance of the sensors were not affected by introduction of the MR_CHIROD in the scanner. Conversely, the MR images (phantom, human) suffered no degradation by the introduction of the MR_CHIROD in the MR scanner. The design and testing of the second generation MR_CHIROD was also been published (148).

**Figure 1:**
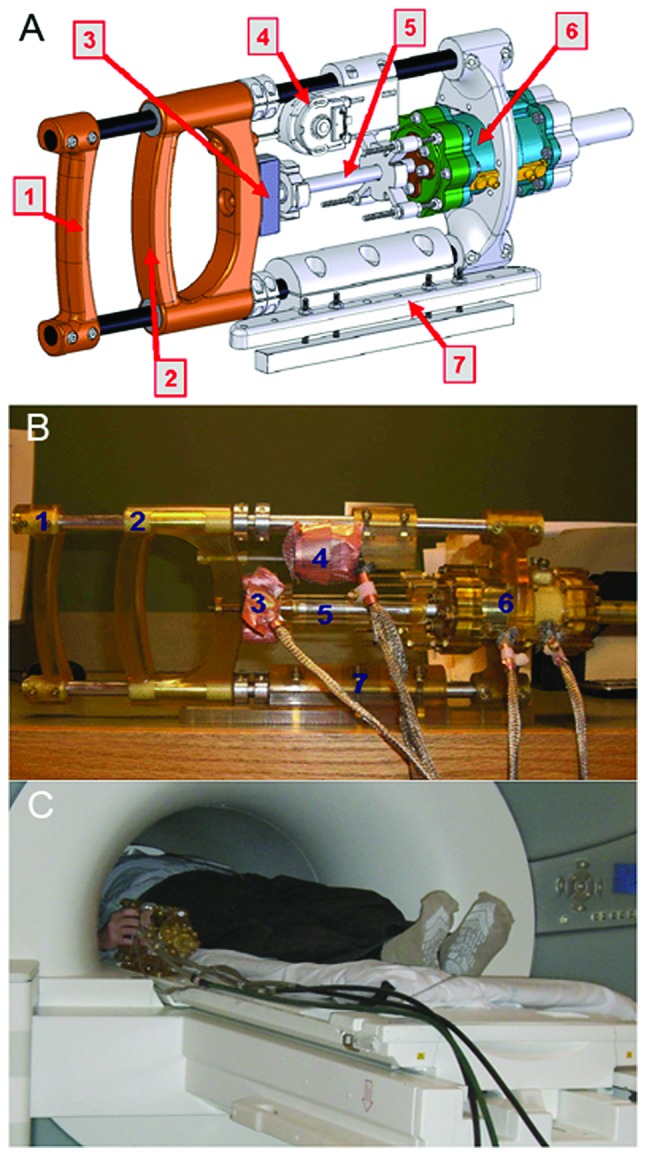
(A) CAD drawing; second-generation MR_CHIROD with linear exercise motion and linear damper. (1) Fixed handle; (2) Moving handle with adjustable range of motion; (3) Force sensor; (4) Position encoder; (5) Piston, connected to moving handle and to damper in ERF fluid; (6) Casing containing the ERF fluid. (7) Base allows firm attachment to the side of the magnet bed and tilt with respect to the vertical axis, maximizing ease of operation. (B) Assembled operational device; (C) MR_CHIROD attached to the scanner while a subject, lying in the magnet, operates it.

**Figure 2:**
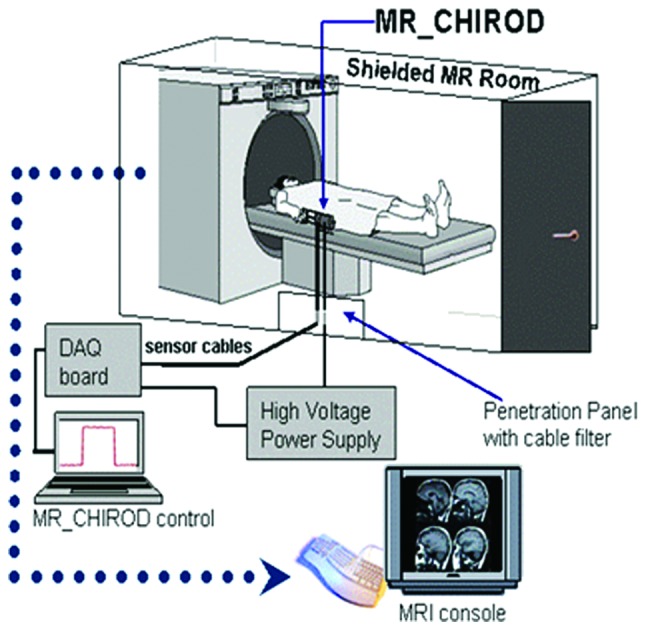
The Magnetic Resonance Compatible Hand-Induced Robotic Device (MR_CHIROD) concept for online brain MRI. The current MR_CHIROD (version 2) is controlled by a data acquisition (DAQ) card and DAQ software on a PC located in the operator room, outside the RF-shielded MR system. This DAQ/PC configuration can be re-engineered into a compact electronic unit. The PC via its A/D and D/A boards collects, stores and visualizes in real-time the encoder and torque measurements. Based on the selected exercise protocol, it also sends the required control voltage to the MR_CHIROD actuators. The PC voltage output is amplified using a very fast, high-voltage power supply and a high-voltage, low-current amplifier circuit board provided by the ERF manufacturer. The damper consists of two electrodes and contains the ERF fluid. The piston (piston shaft drawn in Fig. 1) moves through the ERF fluid with a controlled force of contraction provided by the voltage-controlled variable viscosity of the ERF fluid. A Faraday cage encloses the core of the device, allowing a necessary opening for the movable piston shaft. The negative electrode of the damper (connecting to the negative terminal of the power supply) and the Faraday cage are grounded to the penetration panel of the MR room. A low-pass filter (LPF) is attached to the penetration panel. Sensor readings (force, position) are transmitted through the penetration panel via grounded DSub-9 connectors. The sensor wires are coaxially shielded and grounded to the penetration panel. The sensor readings are used for real-time, closed-loop control of the ERF resistive element. The output from the control loop regulates the voltage output of the power supply, in turn ensuring control of the ERF resistive element and force of contraction.

An assembled MR_CHIROD is shown in Fig. 1. The MR_CHIROD consists of three major subsystems: i) an ERF resistive element, ii) handles and iii) two sensors, include an optical encoder to measure patient-induced motion and a force sensor. Each subsystem includes several components of varying complexity. All components were optimally designed with strength and safety in mind for MR-compatibility and for regular and high-stress testing. The MR_CHIROD is configured to securely attach to the scanner table next to the subject who thus feels no weight. The MR_CHIROD is designed to provide up to 200 N resistive force and to be controlled in real-time (148). Fig. 2 summarizes the online brain MRI concept (95) The MR_CHIROD attaches securely to the scanner table next to the participant, who feels no weight.

## 7. Brain fMRI studies

Relevant published studies (13–15,30,94–96,145–149,153–162) conclude that: i) when used in conjunction with the MR_CHIROD, fMRI data can provide a biomarker for neurological recovery after a stroke; ii) post-stroke cortical and sub cortical reorganization can be assessed with high sensitivity by fMRI; iii) limb dysfunction is related to loss of brain activation; iv) functional recovery after stroke is associated with preservation or restoration of brain activation; v) stroke affects neural connectivity; vi) goal-directed robotic therapy can improve motor abilities in stroke patients and such changes are sustained for at least four months; vii) hand training in chronic stroke patients enhances cortical activation as assessed by fMRI and improves motor performance in a manner consistent with functional plasticity; viii) structural plasticity co-localizes with regions of functional plasticity in the human brain after stroke; and ix) connectivity alterations in motor-related areas are suggestive of functional motor systems reorganization in stroke patients. Although supported by our preliminary data, the last three statements require further validation from studies with large number of participants.

**Figure 3:**
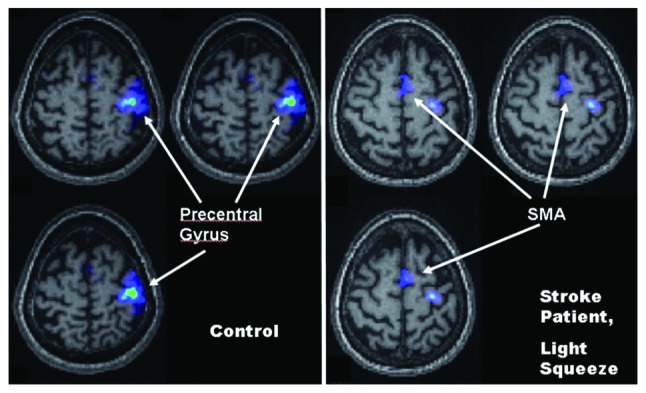
Activation pattern of a 75-year-old stroke patient squeezing at 15% grip strength (right panel) compared to healthy controls (left panel). FMRI activation is superimposed on T1-weighted images. SMC activation is shown at the precentral gyrus; SMA, supplementary motor area.

**Figure 4:**
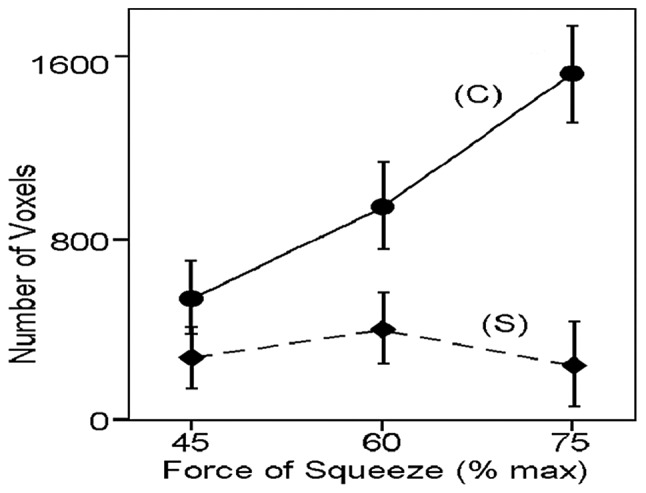
Activated voxels were enumerated for healthy volunteers (controls, C) and stroke patients (S) in the contralateral SMC and increased with force of squeezing in controls. Values are means ± SE. The level of performance was consistently lower in stroke patients than in controls.

Fig. 3 shows data from a 75-year-old stroke patient (2 years after a stroke that affected the left hemisphere). With a 15% effort level squeeze, both SMC and SMA were activated, but the activation in SMC (at the precentral gyrus) was less than in controls. Fig. 4 shows that the canonical curve defining the relationship between brain cortical activation and force of squeezing in controls (C) differs from the shape of the analogous curve in stroke patients (S), which is almost flat. Finally, recently published work suggests that stroke patients exhibit structural plasticity in the same sensorimotor cortical areas that exhibit functional plasticity (94). These results provide the first evidence of structural plasticity co-localized with areas exhibiting functional plasticity in the human brain after stroke.

## 8. Functional cortical plasticity in chronic stroke induced by hand motor training

**Figure 5:**
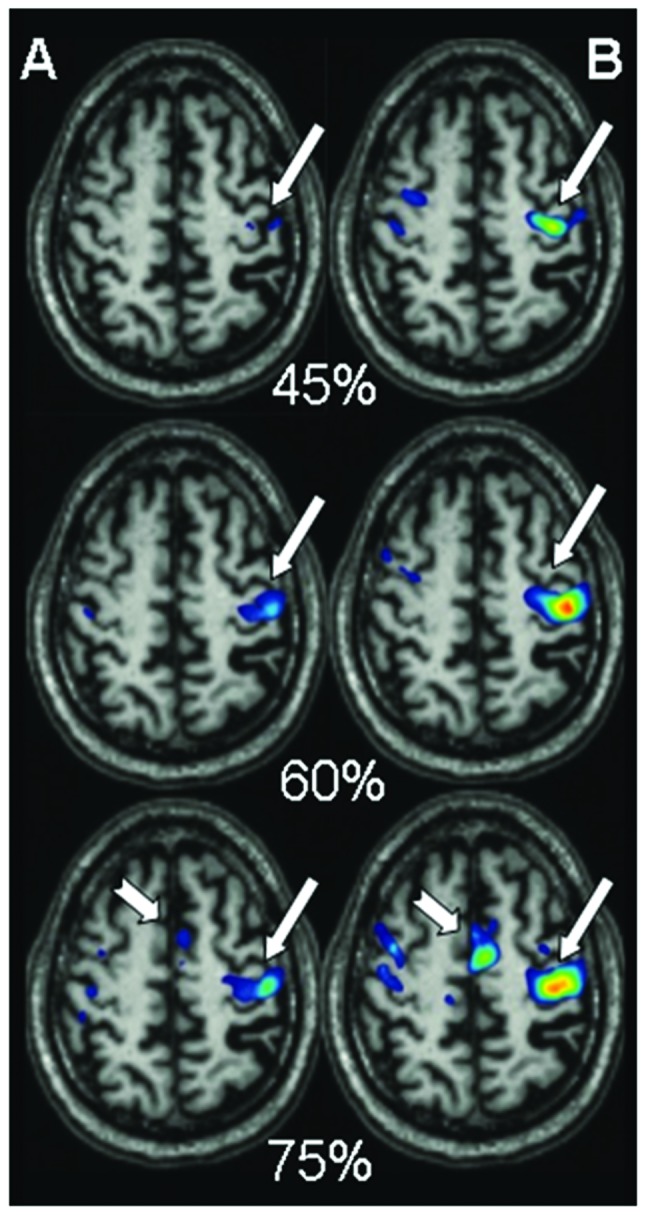
fMRI conducted with MR_CHIROD shows functional cortical plasticity in a chronic stroke patient. (A) Patient performance halfway through training. (B) Patient performance after full 8-wk training. Patient squeezed the MR_CHIROD at 45, 60 and 75% maximum grip force. Activation threshold P<0.05 corrected; activation maps are superimposed on the patient’s T1-weighted anatomical images. SMC activation, longer arrow; SMA activation, shorter arrow). Red color (t-score=10, P<0.0001); Blue color (t-score=4.8, P=0.05).

**Figure 6:**
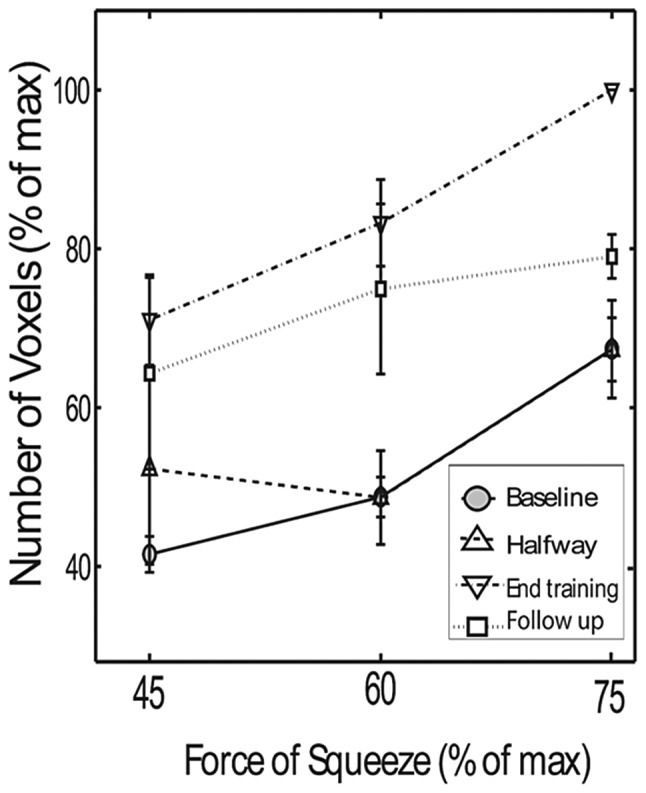
Number of activated voxels in the contralateral SMC as a function of squeezing force in chronic stroke patients. Online mapping was performed at four time-points: baseline (solid line); halfway through training (lower dashed line); at the end of the training (upper dashed line) and 4 weeks after training (dotted line). Note the persistence of increased cortical activation observed during and after the training period.

In a recent study, stroke patients training at home with exercise gel balls (Cando gel hand exercise balls; www.bpp2.com/physical_therapy_products/2932.html) underwent fMRI (95) using the second-prototype MR_CHIROD. Results are shown for a representative patient (63-year-old, right-handed male with subcortical MCA stroke, 4 years post-stroke). The number of activated voxels had increased overall and as a function of effort level at completion of the 8-week training period (Fig. 5). Fig. 6 summarizes results from 5 patients squeezing at three performance levels and over four time-points (baseline, halfway through training, end of training, and at follow-up 4 weeks after completion of training). There were a higher number of activated voxels upon completion of training than at baseline or halfway through training for all three submaximal performance levels. For example, squeezing at 60% effort at the completion of training resulted in 83.25±5.45% activated voxels, compared with 48.74±2.53% at baseline (P<0.0001). Significant behavioral gains were also found at the end of treatment. For example, mean arm motor Fugl-Meyer score at the end of treatment increased from 42±7 at baseline to 55±6 after treatment (P<0.05). Likewise, mean Action Research Arm Test score increased from 37±15 at baseline to 40±14 after treatment (P<0.05). SMC activation with 60% effort squeezing 4 weeks after training completion remained higher (74.94±10.71%) than at baseline (P<0.05) (Fig. 6). A similar trend was observed with 75% effort squeezing, though the comparison to baseline data did not reach statistical significance. These results suggest that the increased SMC activation persists at a reduced degree 4 weeks after training. Importantly, these data demonstrate functional cortical plasticity in chronic stroke accompanied by recovery of motor performance. They also confirm a previous report by Fasoli and colleagues (30) in which chronic stroke patients subjected to goal-directed robotic therapy showed significantly improved motor abilities assessed by traditional motor evaluation; these improvements were sustained 4 months after discharge.

## 9. Significance

Assessing neuroplasticity by means of multiparametric MRI is important for the evaluation of sensorimotor brain networks. Neurological deficit may be better predicted and more precisely characterized by incorporating functional maps of injury assessed with MRI (163). Functional maps can provide insight as to which parts of a system are still functioning, thereby potentially providing new information not be evident from clinical observations alone (97).

Currently, sparse data indicate a relationship between brain activation and functional improvement during therapy suggesting that serial fMRI can be used in predicting the success and optimal duration of therapeutic intervention (108). New studies are required to fully establish the link between changes in fMRI responses and functional outcomes. Then multiparametric MRI combined with a MR-compatible robot would help neurologist to select the most appropriate rehabilitation approach and then fine-tune it based on brain activity. This approach would allow identification of the brain areas that need to be targeted in each individual patient. For example, since the integrity of the corticospinal tract (CST) is a major determinant of motor recovery (164–166), particularly within the first few weeks of recovery (167) and both the extent of structural damage to the CST and subsequent functional reorganization can be observed by MRI (168) the ipsilesional motor cortex might be targeted in patients with largely undamaged ipsilesional motor cortex and CST. In contrast, the contralesional motor cortex might be targeted for therapy in patients with damaged ipsilesional motor cortex and corticospinal tract. Different rehabilitation approaches would likely have differential impacts on the activity of brain areas or networks that mediate spontaneous and compensatory motor recovery in stroke patients. Task-specific training might promote spontaneous motor recovery by normalizing ipsilesional motor cortex activity. In contrast, constraint-induced movement therapy might promote an increase in recruitment of an attentional network.

## 10. Conclusions

The benefit of technology to aid in rehabilitation techniques is now begging to be realized. The Longitudinal multiparametric MRI assessment of brain reorganization in conjunction with MR-compatible robotic devices in chronic stroke is a rapidly evolving field which demonstrates outstanding potential. Stroke rehabilitation is relatively new, having been set back by the longstanding view that lost functions were not recoverable. Robotic devices similar to MR_CHIROD may prove to be valuable in restoring motor performance. The role of robotic therapy is likely to increase as the discrete components of therapeutic intervention become better-understood and robotic assessment techniques are developed in the molecular medicine era.
